# Hunger in the shadow of conflict: analyzing malnutrition and humanitarian challenges in Sudan

**DOI:** 10.1186/s13031-024-00604-6

**Published:** 2024-08-05

**Authors:** Amira Mohamed, Anmar Homeida

**Affiliations:** 1 Sudanese American Physicians Association, Khartoum, Sudan; 2 Montefiore Medical Center, Albert Einstein College of Medicine, Bronx, NY USA; 3 New York University, School of Global Public Health, New York, USA

## Abstract

**Background:**

Conflict has become a global reality, particularly impacting millions of children, with the majority of conflicts occurring in developing nations, where 90% of the world's children reside. The Horn of Africa, especially Sudan, has faced severe conflicts, with the year 2023 witnessing one of the toughest conflicts in the region, resulting in a high number of internally displaced persons and refugees. Children, especially in areas like Darfur, Khartoum, Gezira, and Kordofan, bear the brunt of ongoing large-scale conflicts, facing widespread human rights violations and resource damage.

Before the conflict that began in April 2023, Khartoum was home to numerous children's hospitals, but now only Elbuluk Hospital remains operational, facing a surge in admissions due to displacement and subsequent returns of civilians seeking medical care. Although malnutrition cases have increased, the case fatality rate associated with severe acute malnutrition has doubled from approximately 6% to 12% by March 2024, possibly due to uneven food distribution amid sporadic peace efforts.

**Recommendations:**

Investing in grassroots organizations is crucial for facilitating effective humanitarian aid delivery, as they are uniquely positioned to identify and address local needs promptly and efficiently. Strengthening these organizations enhances their capacity to coordinate aid distribution and provide essential services tailored to regional conditions.

Persistent violations of International Humanitarian Law (IHL) in conflict zones impede humanitarian efforts. Robust collaboration between international and local stakeholders is necessary to uphold and enforce IHL, with a focus on protecting civilian lives and ensuring safe, unhindered access for humanitarian aid while respecting the dignity of all affected individuals.

## Overview of data and context

Conflict has become a pervasive reality globally, impacting millions of children. Most of these conflicts take place in developing nations, which house 90% of the world's children [1]. Africa, especially its Horn region, bears the brunt of high-intensity conflicts [2]. During the year 2023, Sudan has witnessed the toughest conflict in the region with the highest number globally recorded for internally displaced persons- IDPs and refugees [3]. Several areas in Sudan, including Darfur, Khartoum, Gezira, and Kordofan, have witnessed multiple conflicts in the past, however, the recent war that began in April 2023 has resulted in widespread human rights violations and substantial damage to resources. Furthermore, ongoing large-scale conflicts in Darfur, Kordofan, Khartoum, and Gezira persistently endanger the lives of numerous children and adolescents [3, 4].

Although conflict impacts individuals of all ages and genders, women and children are particularly vulnerable [5]. Evidence indicates that more than two-thirds of the world's children reside in conflict-affected regions [6]. Children in these zones suffer both directly and indirectly from the ramifications of conflict [7]. They endure extreme violence, disabilities, hunger, malnutrition, the denial of basic humanitarian needs, and restricted access to health services [6]. Research has also shown that, as a result of ongoing conflicts over recent decades, approximately two million children have been killed, four to five million have been disabled, twelve million have been rendered homeless, over one million have been orphaned, and nearly ten million have suffered psychological trauma [8, 9].

Further evidence highlights that conflict has drastically undermined social structures and cohesion, leading to family and societal breakdowns [10]. Children affected by such disintegration lack social protections and face heightened risks of neglect, abuse, violence, child labor, and trafficking [10]. Moreover, children often witness the horrors of their families and communities fighting, fleeing, being wounded, or dying in conflicts [11]. These profound experiences can severely damage their psychological development, shaping their attitudes toward society, influencing their future relationships, and altering their overall life perspectives.

Before the ongoing war that started in April 2023, Khartoum, Sudan, was home to more than a dozen children’s hospitals that served as primary treatment centers for nearly 730,000 Sudanese children who were suffering from severe acute malnutrition [12, 13]. Today, in a war-stricken Khartoum, only one pediatric hospital remains: Elbuluk Hospital. This small hospital, with no more than 80 inpatient beds, handles cases ranging from neonatal complications to general pediatric emergencies. As the sole pediatric hospital still operational, Elbuluk Hospital has become a crucial data source and a representation of the collapse of Sudan's healthcare system.

Data collected by healthcare providers at Elbuluk Hospital illustrates a significant increase in hospital admissions between August 2023, four months after the war began, and March 2024. The number of admissions nearly doubled from 397 to 834 patients during this period. This surge in admissions can be attributed to the displacement of civilians, particularly during the peak of the conflict in Khartoum around June 2023. Many people were forced to flee their homes, seeking refuge and medical care in other cities [14].

Subsequently, there was a notable return of civilians in waves when the conflict spread to other areas of Sudan, including Wad Madani in January 2024. Some individuals were compelled to return to Khartoum in search of peace and stability once again, leading to another influx of patients at the hospital [14].

While the overall number of malnutrition cases has been increasing, it appears to be slightly behind the surge in hospital admissions at Elbuluk Hospital. However, despite this lag, the case fatality rate associated with severe acute malnutrition (SAM) has doubled from approximately 6% in August 2023 to 12% by March 2024 [14].

This significant increase in the SAM fatality rate may be attributed to several factors. As the situation in the Sudanese capital appeared to stabilize and some civilians returned amidst sporadic peace, the return of food supply chains was uneven [14]. This uneven distribution of food resources likely contributed to an increase in malnutrition incidence, particularly among vulnerable populations such as children suffering from severe acute malnutrition.

### Analysis of malnutrition factors

Sudan ranks among the highest globally in child displacement [15]. Research indicates that displaced children encounter numerous challenges such as inadequate access to basic services, and heightened risks of violence, exploitation, abuse, and trafficking. They are also more susceptible to child labor, child marriage, and family separation, all of which directly endanger their health and safety [16, 17].

The healthcare delivery system in conflict-affected regions remains critically compromised. This is largely due to the destruction of medical facilities, theft of essential medicines and supplies, and disruptions to transportation and curfews, all contributing to substandard healthcare services in these areas. Additionally, intense security concerns lead to a scarcity of healthcare personnel available to provide essential services [18]. Consequently, children in conflict zones often remain untreated and succumb to diseases that are otherwise preventable [19].

Food and nutritional insecurity are another significant concern in conflict-affected areas [20]. Studies have shown that 75% of the world’s stunted and wasted children live in conflict affected areas and their number has gradually increased to 112.1 million [21]. A considerable number of children continue to die from malnutrition in conflict-ridden areas globally. Malnutrition compromises the immune system, increasing susceptibility to lethal diseases such as cholera, pneumonia, and other infections. It also leads to impaired physical and cognitive development among surviving children [22].

Currently, Sudan is experiencing what could be termed the world's worst hunger crisis, with 18 million people, more than a third of the country's population, facing acute food insecurity. This situation is particularly severe in conflict hotspots like the Darfur and Kordofan regions, and Khartoum and Al Jazirah states. These areas have seen a reduction in agricultural output and significant disruptions to trade flows, which together with spiraling prices, have made food increasingly inaccessible to the majority of the population [23]. Children and adolescents in conflict zones endure extreme levels of violence. In 2016, over 1 billion children under 18 years old experienced physical, sexual, or emotional violence, predominantly in African countries like Sudan [24, 25]. Moreover, more than one-third of children living in war zones have suffered from various mental health issues, leading to depression, anxiety, posttraumatic stress disorders, and other behavioral problems [26].

Amidst ongoing conflict, Sudan faces a severe economic crisis characterized by hyperinflation, a weakening currency, and disrupted trade routes, which have compounded pre-existing challenges. These economic disruptions have significantly impaired food production and distribution systems, leading to widespread food insecurity [25].

The direct correlation between the economic downturn and food availability is evident in the scarcity of essential food commodities and soaring food prices, making necessities unaffordable for a large segment of the population. This situation is exacerbated by the displacement of communities due to conflict, which further limits access to food and disrupts local food markets [27].

Malnutrition rates in Sudan have surged because of these compounded issues [28]. The lack of access to a nutritious and adequate diet has led to increased rates of both acute and chronic malnutrition, particularly among vulnerable populations including children, pregnant women, and the elderly. The nutritional crisis is likely to worsen without significant interventions, as the economic instability continues to hinder the effectiveness of aid and governmental support systems [29, 30].

Food accessibility during fighting the nexus between conflict and climate change, and the impact on local food markets and supply chains are critical aspects that significantly influence food insecurity, especially for children and women, defined as the limited or uncertain ability to acquire adequate and nutritious food in socially acceptable ways [31].

### Link to humanitarian situation and international humanitarian law

Humanitarian access in Sudan remains a critical issue for various international stakeholders, including the United States, which has urged the UN Security Council to permit aid deliveries through Chad. The situation in Sudan deteriorated notably following the escalation of conflict in April 2023 [32]. In the initial month of hostilities, over six hundred fatalities were reported, with significant damage inflicted on hospitals and critical infrastructure. By August 2023, the United Nations described the escalating violence in Sudan as "spiraling out of control," highlighting the exodus of refugees and a collapsing healthcare system that heightened the risk of disease outbreaks. The displacement crisis is particularly alarming due to the regional instability, with Sudan sharing borders with volatile states like the Central African Republic, Chad, Eritrea, Ethiopia, Libya, and South Sudan. The UN’s chief of Humanitarian and Emergency Relief labeled the situation in Sudan as "one of the worst humanitarian nightmares in recent history" [32].

On March 8, 2024, the UN Security Council adopted a resolution demanding an immediate halt to the violence in Sudan. Shortly thereafter, the Sudanese Armed Forces (SAF) entered indirect talks with the Rapid Support Forces (RSF), mediated by Libya and Turkey. However, these negotiations faltered on different occasions and were massively failed due to the lack of commitment of conflict factions to adhere to the International Humanitarian Law(IHL) and put the benefits of civilians first to provide safe corridors for humanitarian aid [33].

Malnutrition indicators play a critical role in understanding and responding to broader humanitarian emergencies in Sudan. They provide key insights into the nutritional status of affected populations, highlight the strain on health systems, guide targeted humanitarian interventions, emphasize the impact on vulnerable groups, and serve as proxy measures to assess the overall severity of the crisis [11].

### Implications and recommendations

There is an imperative need for sustained and equitable access to food aid and nutritional support to mitigate the degradation of health outcomes among populations affected by the conflict in Sudan. Addressing food insecurity and malnutrition should be prioritized to lessen the adverse effects of the ongoing crisis on vulnerable groups and families within the region, and guard against the risk of a nationwide famine [34, 35].

To effectively combat food shortages, it is essential to concentrate on improving local agricultural practices. Initiatives that support local farmers and enhance community-based agricultural programs are crucial for increasing food availability. This approach not only aids in stabilizing food prices but also secures a resilient food supply chain capable of withstanding socio-economic disturbances [36].

Furthermore, malnutrition, a severe consequence of food shortages, necessitates immediate and localized interventions. Decentralizing the management and treatment of malnutrition allows community-based nutritional programs to play an essential role. Managed at the local level, these programs improve the accessibility and efficiency of healthcare services, providing tailored solutions that meet the specific needs of the community [37].

In conflict zones, there is often a significant disruption of healthcare services, making the reconstruction of local healthcare infrastructure a priority. Investing in local healthcare facilities and the professional development of medical personnel are critical steps toward revitalizing healthcare delivery. Such initiatives ensure the sustainability of health services and enhance community resilience in responding to health crises [38].

Addressing the immediate challenges of food insecurity also calls for innovative economic strategies such as cash transfers. These provide families with the flexibility to address their unique needs while stimulating local economies. Moreover, improving the logistics of aid delivery is crucial to ensure that assistance is not only delivered promptly but also reaches the intended recipients efficiently [39, 40].

Furthermore, investing in grassroots organizations is paramount for facilitating effective humanitarian aid delivery. These organizations, deeply embedded within their communities, are uniquely positioned to identify, and address local needs promptly and efficiently. Their pivotal role in ensuring that aid reaches the most vulnerable populations cannot be overstated. Strengthening these organizations enhances their capacity to coordinate aid distribution, engage with community members, and provide essential services tailored to the specific conditions of the region [41].

Finally, persistent violations of International Humanitarian Law (IHL) in conflict zones significantly impede humanitarian efforts. Robust collaboration between international and local stakeholders to uphold and enforce IHL is essential, with an emphasis on protecting civilian lives and ensuring safe, unhindered access for humanitarian aid. Addressing these challenges involves not merely providing aid but ensuring it is delivered in a manner that respects and safeguards the dignity of all individuals affected by conflict [42].

## Conclusion

The dreadful condition in Sudan requires a concerted effort from all stakeholders to prioritize the protection of civilians and ensure effective delivery of humanitarian aid. By localizing aid interventions and engaging community assets, we can hope to not only alleviate the current suffering but also lay down the groundwork for sustainable recovery and resilience.

Addressing the complex challenges of food insecurity and health deterioration in conflict zones like Sudan necessitates a multifaceted approach. Sustained and equitable access to food aid and nutritional support is critical to prevent further health crises among affected populations. Enhancing local agricultural practices and investing in grassroots organizations are essential to ensure a stable food supply and efficient aid delivery. Decentralizing the management of malnutrition and rebuilding local healthcare infrastructure are pivotal in enhancing the resilience and sustainability of health services. Additionally, innovative economic solutions such as cash transfers and improved logistics are crucial for empowering affected families and ensuring that aid reaches those in dire need promptly and effectively. Moreover, upholding International Humanitarian Law is imperative to protect civilian lives and ensure that humanitarian efforts are conducted with respect and dignity. Together, these strategies underscore the importance of a coordinated, community-focused response that leverages local strengths and international support to mitigate the impacts of conflict and build towards a sustainable future.

This narrative isn't just a plan of action—it's a call to safeguard the future of Sudan's most vulnerable populations amid one of the most challenging humanitarian crises of our time.

## Data Availability

No datasets were generated or analysed during the current study.
